# Promoting Pancreatic Fistula Healing After Pancreaticoduodenectomy Through Internal Drainage of Pancreatic Juice Into the Jejunum

**DOI:** 10.1002/wjs.12679

**Published:** 2025-07-12

**Authors:** Yoshihiro Miyazaki, Shinji Hashimoto, Osamu Shimomura, Hiromitsu Nakahashi, Manami Doi, Kazuhiro Takahashi, Jaejeong Kim, Shuntaro Tsukamoto, Kinji Furuya, Yohei Owada, Koichi Ogawa, Yoshimasa Akashi, Tsuyoshi Enomoto, Tatsuya Oda

**Affiliations:** ^1^ Department of Gastrointestinal and Hepato‐Biliary‐Pancreatic Surgery Institute of Medicine University of Tsukuba Tsukuba Japan

**Keywords:** drain management, fistulography, jejunal drainage, pancreaticoduodenectomy (PD), postoperative pancreatic fistula (POPF)

## Abstract

**Background:**

Postoperative pancreatic fistula (POPF) is a significant complication of pancreaticoduodenectomy (PD), leading to severe morbidity and prolonged hospitalization. Drain‐tract‐targeted management and fistulography are important in the treatment of clinically relevant POPF. However, the healing process for POPF remains unclear.

**Methods:**

A retrospective analysis was conducted on 63 patients who underwent pancreaticojejunostomy at our institution between 2017 and 2022 and underwent fistulography at least twice. The patients were classified by drain contrast type into (1) the fistulous tract (FT) group (*n* = 12), where only the fistula was contrasted or (2) the fluid collection (FC) group (*n* = 51), where the FC was connected to the drain fistula. The postoperative outcomes, including POPF resolution and drainage duration, were compared between the two groups.

**Results:**

The incidence of POPF (International Study Group on Pancreatic Fistula grades BL, B, and C) was comparable between the FT (91.6%) and FC (94.1%, *p* = 0.53) groups. More patients in the FC group (60.8%) achieved POPF resolution through drainage into the jejunum than in the FT group (16.7%, *p* < 0.001). The median drainage duration was significantly shorter in the FT group (22.5 days; interquartile range [IQR], 16.1–24.3) than in the FC group (28.0 days; IQR, 21.5–33.5; *p* = 0.011). More drain exchanges were required in the FC group (median, 4; IQR, 3.0–5.0) than in the FT group (median, 3; IQR, 2.0–3.3; *p* = 0.01). All patients in the jejunal drainage group followed the same course: after fistulography showed that contrast was flowing into the jejunum, the drainage fluid decreased, and the drains were eventually removed. Readmission, intra‐abdominal abscess, or re‐drainage after drain removal was not observed.

**Conclusion:**

Internal drainage into the jejunum significantly improved POPF healing after PD. This study highlights the importance of monitoring and managing drainage patterns using fistulography.

## Graphical abstract

Drain‐tract‐targeted management and fistulography are important in the treatment of Postoperative pancreatic fistula (POPF). Internal drainage into the jejunum significantly improved POPF healing after pancreaticoduodenectomy.

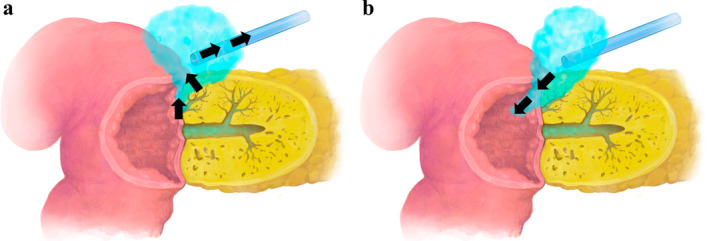

## 1 Introduction

Despite recent advances in surgical techniques and postoperative management, the 90‐day mortality rate for patients after pancreaticoduodenectomy (PD) remains approximately 2%, making it one of the most invasive and dangerous procedures [1, 2]. Postoperative pancreatic fistula (POPF) is one of the most significant complications following PD, with an incidence ranging from 5% to 26%, depending on patient characteristics and institutional experience. Despite ongoing research, POPF remains one of the most difficult complications to treat [1, 2]. POPF is associated with severe complications such as intraperitoneal abscesses and hemorrhage, often resulting in prolonged hospitalization and, in severe cases, mortality. These challenges make POPF one of the most critical issues affecting PD outcomes.

In most medical centers, prophylactic drains are placed around the pancreaticojejunostomy site for patients at high risk of POPF. Amylase levels in the drain fluid have been reported as useful indicators for identifying patients who may benefit from early prophylactic drain removal after PD [3]. Indeed, some studies suggest that the early removal of prophylactic drains reduces the incidence of clinically significant POPF [4]. However, these findings remain controversial, and definitive evidence is lacking. Moreover, the pathophysiology of POPF and its healing process have not been fully elucidated.

Recent literature highlights the potential of “drain‐tract‐targeted management” and fistulography as key elements in POPF management [5, 6, 7, 8, 9]. Fistulography offers a unique opportunity to visualize and categorize the patterns of pancreatic leakage, providing insights into the severity and progression of POPF. However, the relationship between fistulography findings and the healing process of POPF remains poorly understood.

At our institution, the management of POPF emphasizes prophylactic fistulography and active drain replacement rather than early drain removal. Drain removal is performed only after confirming POPF resolution. This unique management approach, coupled with detailed fistulography analysis, provides valuable insights into the healing process of POPF. This study aimed to investigate the relationship between fistulography drainage patterns and POPF outcomes, particularly the role of pancreatic juice drainage into the jejunum in facilitating fistula healing.

## 2 Materials and Methods

### 2.1 Study Design and Patient Selection

This retrospective study included 258 consecutive patients who underwent PD in the Department of Gastrointestinal and Hepato‐Biliary‐Pancreatic Surgery at the University of Tsukuba Hospital between 2017 and 2022. During this study period, pancreatic reconstruction was performed using pancreaticojejunostomy with the Blumgart method and anastomosis of the main pancreatic duct to the jejunal mucosa. Patients were excluded if they underwent pancreaticojejunostomy using the Blumgart method with the addition of inter‐anastomotic drainage (*n* = 95) [10]. Additionally, patients were excluded if they had undergone only one (*n* = 13) or no (*n* = 87) fistulography procedure. In patients with a low amylase level in the drainage fluid and no suspicion of POPF, the drain was removed without fistulography. A total of 63 patients met the inclusion criteria and were analyzed in this study (Figure S1: Figure (a)). Standard demographic and clinicopathological data, including age, sex, tumor‐specific characteristics, and drain contrast images after PD, were collected. Written informed consent was obtained from all patients before surgical intervention. This study was approved by the Institutional Review Board of the University of Tsukuba Hospital (R01‐030).

### 2.2 Surgical Procedure

Subtotal stomach‐preserving PD was performed in all cases except those following distal gastrectomy. In cases of prior distal gastrectomy, the procedure aimed to spare as much of the stomach as possible. During pancreatic head resection and lymph node dissection, the nerve plexus around the proper hepatic, common hepatic, and superior mesenteric arteries were circumferentially preserved to prevent aneurysm rupture after POPF [11]. Stomach resection was performed 4 cm from the pyloric ring. Consequently, reconstruction was performed using the Child method, which involved end‐to‐side pancreaticojejunostomy, hepaticojejunostomy, and gastrojejunostomy on the same jejunal limb [12]. Pancreatic anastomosis involved duct‐to‐mucosa anastomosis via continuous suturing and the modified Blumgart method, which involved clamping the pancreatic parenchymal stump with the jejunal seromuscular layers using horizontal mattress‐type penetration sutures [13]. An external stent tube was used in all cases except for those involving hard pancreatic tissue. As in previously reported papers, two 20–22 Fr silicone tubes (Fuji Systems Co., Tokyo, Japan) were prophylactically placed for drainage on the cranial and caudal sides of the pancreaticojejunostomy [12, 13].

### 2.3 Postoperative Drain Management

The postoperative drainage management workflow is shown below (Figure S1: Figure (b)). The volume of fluid drained from the cranial and caudal side drains was quantified daily, and amylase levels were monitored on postoperative days (PODs) 1, 3, and 5. The higher of the two drain amylase values was recorded and used in the analysis. POPF diagnoses were made in accordance with the criteria established by the International Study Group on Pancreatic Fistula (ISGPF) [14]. Patients with drainage amylase values greater than 3 times the serum value, those requiring drainage for more than 3 weeks, and those with low drainage amylase values but infectious drainage were diagnosed with POPF. Initial drain contrast imaging and drainage tube exchange were conducted between PODs 4 and 7, depending on the drain amylase level or the presence of infection in the drain fluid. If the following criteria were met on PODs 5–7, the drainage tubes were removed. The criteria for removal of the drainage tubes were (1) amylase level of the drainage fluid < 1000 IU/mL, (2) absence of infection in the drainage fluid, (3) reduction in drainage fluid volume, and (4) improvement in systemic inflammation. If these criteria were not met, the drainage tubes were replaced. In cases where the amylase concentration in the effluent was low and the color and viscosity of the effluent indicated pancreatic fluid contamination, drainage tubes were replaced. The type of drain was selected from either a silicone or Neraton catheter, and the optimal thickness was chosen from 10 to 20 Fr according to the size of the fluid collection. The new drainage tubes were maintained openly, and contaminated fluids were intermittently flushed with normal saline two to three times daily on average. The drainage tubes were subsequently removed once POPF resolved. If the POPF did not improve, fistulography was performed, and the drainage tube was replaced at the optimal site once or twice weekly.

### 2.4 Fistulography and Radiological Classification

**FIGURE 1:**
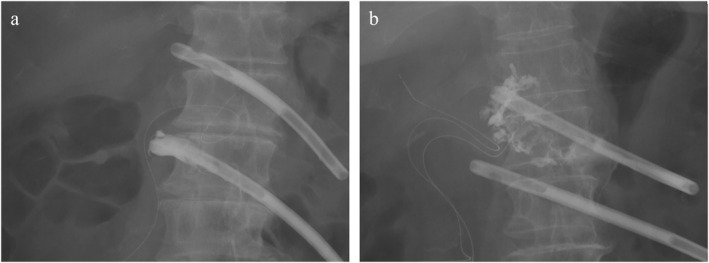
Radiologically classified initial drain contrast imaging into two types: (a) fistulous tract (FT) group (only fistula contrasted) and (b) fluid collection (FC) group (fluid collection connected to the drain fistula).

Hepato‐biliary‐pancreatic surgeons performed fistulography in an X‐ray fluoroscopy room. Plain radiographs of the abdomen were obtained with the patient in the supine position to identify the radiopaque drain tip. Following the replacement of the abdominal drain with an 8 Fr Nelaton catheter (TERUMO, Tokyo, Japan) and a guidewire (Safe‐T J Curved, COOK, USA) with a diameter of 0.035 cm, a 20‐mL volume of water‐soluble contrast agent was injected into the drain. In our department, drain management was conducted in accordance with a consensus reached at a surgical meeting. Imaging and drain replacements were performed weekly until the drains were removed. Fistulography findings were classified into two groups: (1) a fistulous tract (FT) group (only the fistula was contrasted) and (2) a fluid collection (FC) group (fluid collection connected to the fistula was observed) (Figure 1). This classification was similar to the published literature [7, 8, 9]. If both FC and FT were observed, they were included in the FC group. In the presence of FC, drainage was considered poor, and a new drain was placed at the site of best drainage using a guidewire. Radiological and clinical data, including the number of drain exchanges, duration of drainage, and hospital stay, were examined in this study. Postoperative complications were graded according to the Clavien–Dindo classification system [15]. Postoperative hemorrhage was diagnosed in accordance with the International Study Group of Pancreatic Surgery criteria [16].

### 2.5 Statistical Analysis

All calculations were performed using EZR (Saitama Medical Center, Jichi Medical University, Saitama, Japan), a graphical user interface for R (R Foundation for Statistical Computing, Vienna, Austria) [17]. Categorical variables were analyzed using a chi‐square or Fisher's exact test, as appropriate, and presented as numbers with percentages. Continuous variables were analyzed using the Wilcoxon rank‐sum test and expressed as means with standard deviations or medians with interquartile ranges (IQRs) depending on their distribution. All statistical analyses were two‐tailed, and *p* values < 0.05 were considered statistically significant.

## 3 Results

### 3.1 Patient Characteristics

The study included 63 patients who met the inclusion criteria and were categorized into two groups based on fistulography findings: the FT (*n* = 12) and FC groups (*n* = 51) (Figure 1). Table 1 summarizes the patients' demographic and clinicopathological data. No significant differences were observed between the two groups in median age (FT: 73.0 vs. FC: 70.0 years, *p* = 0.22), sex distribution (FT: 41.7% vs. FC: 29.4% female, *p* = 0.50), or patients with the American Society of Anesthesiologists physical status score ≥ 3 (FT: 66.7% vs. FC: 43.1%, *p* = 0.34). Patients with soft pancreas comprised 83.3% in the FT group and 96.1% in the FC group (*p* = 0.16). The median main pancreatic duct diameter (FT: 3.0 vs. FC: 4.0 mm, *p* = 0.47), operative time (FT: 452 vs. FC: 422 min, *p* = 0.63), and blood loss (FT: 626 vs. FC: 526 mL, *p* = 0.68) were similar between the groups.

**TABLE 1:** The demographic data, pathological details, and intraoperative parameters in the FT and FC groups.

	Fistulous tract (*n* = 12)	Fluid collection (*n* = 51)	*p* value
Age, years (IQR)	73.0 (68.8–78.5)	70.0 (68.0–75.0)	0.22
Sex, female, *n* (%)	5 (41.7)	15 (29.4)	0.50
Body weight, kg (IQR)	62.4 (57.4–68.3)	60.2 (53.0–66.7)	0.42
Height, cm (IQR)	160 (151.7–169.1)	159 (154.6–165.6)	0.88
ASA‐PS ≧ 3, *n* (%)	8 (66.7)	22 (43.1)	0.34
Serum albumin, g/dL (IQR)	4.1 (3.9–4.3)	4.1 (3.8–4.4)	0.99
Diabetes mellitus, *n* (%)	4 (66.7)	15 (37.5)	0.21
Diagnosis, *n* (%)			0.89
BTC	4 (33.3)	18 (35.3)	
PDAC	3 (25.0)	7 (13.7)	
IPMN	3 (25.0)	9 (17.6)	
Ampullary cancer	1 (8.3)	10 (19.6)	
NEN	1 (8.3)	4 (7.8)	
Others	0	3 (6.0)	
Pancreas parenchyma, soft, *n* (%)	10 (83.3)	49 (96.1)	0.16
MPD diameter, mm (IQR)	3.0 (3.0–3.0)	4.0 (3.0–5.0)	0.47
Operation time, min (IQR)	452 (418–490)	422 (368–479)	0.63
Blood loss, mL (IQR)	626 (430–978)	526 (359–835)	0.68

Abbreviations: ASA‐PS, American Society of Anesthesiologists‐Physical Status; BTC, biliary tract cancer; FC, fluid collection; FT, fistulous tract; IPMN, intraductal papillary mucinous neoplasm; IQR, interquartile range; MPD, main pancreatic duct; NEN, neuroendocrine neoplasm; PDAC, pancreatic ductal adenocarcinoma.

### 3.2 POPF and Complications

The postoperative data related to POPF are shown in Table 2. Clinically relevant POPF (ISGPF grades B and C) occurred in 58.3% and 74.5% of the patients in the FT and FC groups (*p* = 0.27). Postoperative hemorrhage requiring intervention was observed in 0% of FT patients compared to 5.9% of patients in the FC group (*p* < 0.001). The initial drain exchange procedure was conducted on POD 6 (IQR, 5.0–7.0) in both the FT and FC groups (*p* = 0.89). The median number of drain exchanges was significantly higher in the FC group (4; IQR, 3.0–5.0) than in the FT group (3; IQR, 2.0–3.3; *p* = 0.01). The duration of drainage was significantly longer in the FC group (28.0 days; IQR, 21.5–33.5) compared to the FT group (22.5 days; IQR, 16.1–24.3; *p* = 0.011). Postoperative hospitalization was 20.0 days (IQR, 19.0–26.0) in the FT group and 24.0 days (IQR, 21.0–31.0) in the FC group, with no statistically significant difference (*p* = 0.14).

**TABLE 2:** Postoperative data related to postoperative pancreatic fistula in FT and FC groups.

	Fistulous tract (*n* = 12)	Fluid collection (*n* = 51)	*p* value
Serum AMY on POD1, IU/L (IQR)	456 (304–984)	374 (281–667)	0.63
Serum AMY on POD3, IU/L (IQR)	53 (26–129)	79 (50–152)	0.16
Serum AMY on POD5, IU/L (IQR)	26 (19–54)	43 (28–84)	0.066
D‐AMY on POD1, IU/L (IQR)	3862 (1658–12874)	4451 (1726–13553)	0.79
D‐AMY on POD3, IU/L (IQR)	255 (69–896)	659 (196–2316)	0.13
D‐AMY on POD5, IU/L (IQR)	68 (57–217)	230 (53–470)	0.39
C–D grade, *n* (%)			0.69
3a	4 (66.7)	16 (31.4)	
4	0	3 (5.9)	
ISGPF grade, *n* (%)			0.53
BL	4 (33.3)	10 (19.6)	
B	7 (58.3)	35 (68.6)	
C	0	3 (5.9)	
Postoperative hemorrhage, *n* (%)	0	3 (5.9)	< 0.001
Fist drain exchange, POD (IQR)	6.0 (5.0–7.0)	6.0 (5.0–7.0)	0.89
Number of drain exchanges (IQR)	3 (2.0–3.3)	4 (3.0–5.0)	0.01
Healed with tract, *n* (%)	10 (83.3)	11 (21.6)	< 0.001
Healed with jejunal drainage, *n* (%)	2 (16.7)	31 (60.8)	< 0.001
Duration of drainage, days (IQR)	22.5 (16.0–24.3)	28.0 (21.5–33.5)	0.011
POHS, days (IQR)	20 (19–26)	24 (21–31)	0.14

Abbreviations: AMY, amylase; C–D, Clavien–Dindo; D‐AMY, drain amylase; IQR, interquartile range; ISGPF, international study group of pancreatic fistula; IVR, interventional radiology; POD, postoperative day; POHS, postoperative hospital stay.

### 3.3 The Healing Process of POPF

**FIGURE 2:**
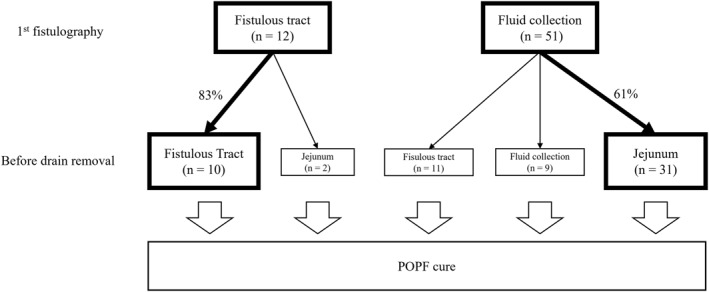
Healing process of postoperative pancreatic fistula (POPF).

**FIGURE 3:**
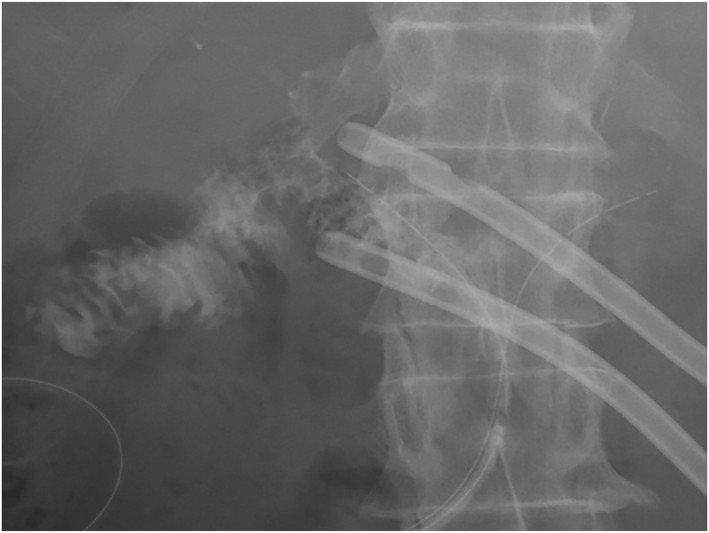
Radiographic image showing drainage into the jejunum.

The healing process of POPF differed between the two groups (Figure 2). In all cases, the drain was confirmed to be close to the pancreaticojejunostomy site, and no cases of poor drainage were observed due to unintentional drain repositioning. In the FC group, FC was located in the area immediately adjacent to the pancreaticojejunostomy, near the drain. Among the 12 patients initially classified as having FT, 10 (83.3%) achieved resolution with only the fistulous tract visible, whereas two patients (16.7%) exhibited resolution with drainage into the jejunum (Figure 3). The contrast agent spontaneously flowed into the jejunum, followed by intestinal peristalsis toward the anus. Conversely, in the FC group, 11 (21.6%) of the 51 patients showed disappearance of the FC and persistence of the FT on the last fistulography. In 31 (60.8%) of the 51 patients, the FC gradually decreased in size, and the last fistulography showed drainage into the jejunum. Nine patients (17.6%) in the FC group exhibited fluid collection around the FT before resolution. The duration of drainage was 22.5 days (IQR, 16.1–24.3) in the FT group and 28.0 days (IQR, 21.5–33.5) in the FC group (*p* = 0.011). Therefore, patients in the FT group had fewer drain changes and were cured in a shorter period than those in the FC group. Severe POPF with FC required more frequent drain exchanges and longer treatment times; however, the final contrast showed drainage into the jejunum, and POPF was cured.

### 3.4 Significance of Jejunal Drainage

Subsequently, the significance of the jejunal drainage was determined. Within the FC group, patients were further stratified based on the presence or absence of jejunal drainage (JDG group: jejunal drainage observed, *n* = 31; non‐JDG group: no jejunal drainage observed, *n* = 20). Patient demographics, operative factors, and postoperative outcomes for the JDG group and non‐JDG group are detailed in Table 3. No significant differences were observed in age, sex, pancreatic parenchymal texture, or main pancreatic duct diameter between the two groups. No patient exhibited jejunal drainage at the first fistulography. The first drain exchange was performed on 6.5 POD (5.0–9.0) in the non‐JDG group and on 6.0 POD (IQR, 5.0–7.0) in the JDG group (*p* = 0.12). The duration from surgery to the first jejunal drainage was 14.0 days (IQR, 10.3–18.0) in the JDG group. The duration of drainage was significantly longer in the JDG group (median, 31.0 days; IQR, 22.0–35.5) compared with the non‐JDG group (27.0 days; IQR, 20.8–29.0, *p* = 0.048). The postoperative hospitalization was significantly longer in the JDG group (25.0 days; IQR, 23.0–34.0) compared to the non‐JDG group (21.0 days; IQR, 18.8–26.0; *p* = 0.007). Patients in the JDG group required significantly more drain exchanges (median, 5; IQR, 4.0–6.0) compared to those in the non‐JDG group (median, 3; IQR, 2.0–4.0; *p* < 0.001). The duration of drainage was correlated with the number of drain changes. All patients in the JDG group followed the same course: after fistulography showed that contrast flowed into the jejunum, the drainage fluid decreased, and the drains were eventually removed. Readmission, intra‐abdominal abscess, or re‐drainage after drain removal was not observed. Therefore, these results showed that patients in the JDG group had more severe POPF than those in the non‐JDG group, and the severe POPF was cured after the contrast agent flowed into the jejunum during fistulography.

**TABLE 3:** Comparison of patient background, surgical and postoperative factors with and without drainage into the jejunum.

	Non‐JDG (*n* = 20)	JDG (*n* = 31)	*p* value
Age, years (IQR)	70.0 (66.5–75.0)	70.0 (68.0–75.5)	0.51
Sex, female, *n* (%)	6 (30)	9 (29)	1
Body weight, kg (IQR)	58.1 (51.8–63.1)	61.6 (55.3–69.7)	0.22
Height, cm (IQR)	160.5 (154.1–167.1)	158.8 (155.3–162.7)	0.6
ASA‐PS ≧ 3, *n* (%)	9 (45.0)	13 (41.9)	0.60
Serum albumin, g/dL (IQR)	4.0 (3.4–4.2)	4.2 (4.1–4.4)	0.011
Diabetes mellitus, *n* (%)	6 (35.3)	9 (39.1)	1
Pancreas parenchyma, soft, *n* (%)	20 (100.0)	29 (93.5)	0.51
MPD diameter, mm (IQR)	4.0 (3.0–6.0)	4.0 (3.0–4.5)	0.24
Diagnosis, *n* (%)			0.71
BTC	6 (30)	12 (39)	
PDAC	3 (15)	4 (13)	
IPMN	3 (15)	6 (19)	
Ampullary cancer	4 (20)	6 (19)	
NEN	3 (15)	1 (3)	
Others	1 (5)	2 (6)	
Operation time, min (IQR)	406 (355–474)	456 (382–488)	0.29
Blood loss, mL (IQR)	460 (340–630)	605 (383–1005)	0.13
Serum AMY POD1, IU/L (IQR)	316 (271–491)	434 (309–816)	0.12
Serum AMY POD3, IU/L (IQR)	81 (62–155)	73 (47–141)	0.30
Serum AMY POD5, IU/L (IQR)	54 (28–87)	43 (27–78)	0.73
D‐AMY POD1, IU/L (IQR)	4730 (824–12551)	4451 (2040–13658)	0.56
D‐AMY POD3, IU/L (IQR)	419 (174–1711)	744 (313–2493)	0.20
D‐AMY POD5, IU/L (IQR)	100 (27–285)	308 (134–614)	0.052
C–D grade, *n* (%)			0.077
3a	4 (20)	12 (38.7)	
4	0	3 (9.7)	
ISGPF grade, *n* (%)			0.49
BL	4 (20)	6 (19)	
B	14 (70)	21 (68)	
C	0	3 (9.7)	
Postoperative hemorrhage, *n* (%)	0	3 (9.7)	< 0.001
Fist drain exchange, POD (IQR)	6.5 (5.0–9.0)	6.0 (5.0–7.0)	0.12
Number of drain exchanges (IQR)	3.0 (2.0–4.0)	5.0 (4.0–6.0)	< 0.001
First jejunum drainage, POD	NA	14.0 (10.3–18.0)	NA
Duration of drainage, days (IQR)	27.0 (20.8–29.0)	31.0 (22.0–35.5)	0.048
POHS, days (IQR)	21.0 (18.8–26.0)	25.0 (23.0–34.0)	0.007

Abbreviations: AMY, amylase; ASA‐PS, American Society of Anesthesiologists‐Physical Status; BTC, biliary tract cancer; C–D, Clavien–Dindo; D‐AMY, drain amylase; FC, fluid collection; FT, fistulous tract; IPMN, intraductal papillary mucinous neoplasm; IQR, interquartile range; ISGPF, international study group of pancreatic fistula; IVR, interventional radiology; MPD, main pancreatic duct; NEN, neuroendocrine neoplasm; PDAC, pancreatic ductal adenocarcinoma; POD, postoperative day; POHS, postoperative hospital stay.

## 4 Discussion

**FIGURE 4:**
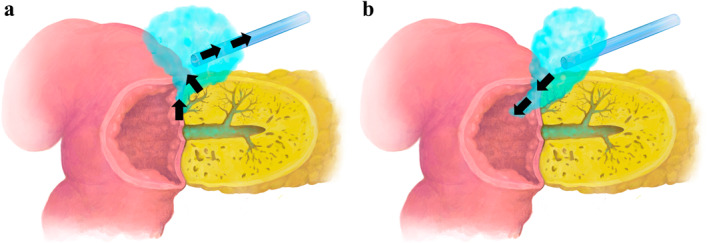
Illustration of POPF drainage and internal drainage into the jejunum in a cross‐section of Blumgart's pancreaticojejunal anastomosis: (a) Pancreatic juice leaking from the pancreaticojejunostomy is collected through the drain. (b) Pancreatic juice leaking from the anastomosis is drained into the jejunum.

This study investigated the role of pancreatic fluid drainage into the jejunum during the healing process of POPF after PD (Figure 4). This present study revealed that jejunal drainage was a significant factor in promoting POPF resolution, highlighting the importance of fistulography and active drain management in postoperative care.

The results of this study complement existing studies that emphasize the importance of drain‐tract‐targeted management and the utility of fistulography in managing POPF [7, 8, 9]. Although risk factors for clinically relevant POPF have been reported, their clinical impact on the outpatient management of pancreatic leakage is limited. Callery et al. proposed a fistula risk score to predict the risk of clinically relevant POPF after PD [1, 18]. This score is based on multiple factors, including pancreatic parenchymal texture, disease pathology, pancreatic duct size, and intraoperative blood loss, and has a validated predictive ability using independent prospective data from multiple centers. However, the pathophysiological processes underlying pancreatic leakage and its treatment have rarely been discussed. Prior research has demonstrated that fistulography provides valuable insights into the severity and progression of POPF [7, 8, 9]. However, the healing process was not addressed. This study found that jejunal drainage was a critical factor in the resolution of POPF and facilitated its healing. All patients in the JDG group followed the same course: after fistulography showed that contrast flowed into the jejunum, the drainage fluid decreased, and the drains were eventually removed. Therefore, these results showed that patients in the JDG group had more severe POPF than those in the non‐JDG group, and the severe POPF was cured after the contrast flowed into the jejunum during fistulography. This current study extends these findings by systematically analyzing the impact of drainage patterns on POPF outcomes, specifically the role of jejunal drainage. Unlike previous studies that focused on early drain removal as a preventive strategy [3] or postulated that prophylactic drains were unnecessary for PD [19], this study emphasizes the active use of fistulography to guide drain management, ensuring that drains should be removed only after POPF resolution is confirmed.

The observed differences in drainage patterns and their correlation with healing also addressed concerns raised in earlier studies regarding the potential risks of jejunal communication. Although it has been suggested that communication between the peritoneal cavity and the jejunum may lead to complications such as infection and intestinal fluid reflux [9], the results of this study demonstrate that controlled jejunal drainage is a key step in resolving POPF rather than exacerbating it. The published report has successfully treated refractory POPF by exploiting the fact that an internal fistula from the pancreaticojejunostomy to the jejunum leads to a cure for POPF [20].

This study had some limitations. As this was a single‐center retrospective analysis, the findings may not be generalizable to other institutions or patient populations. Additionally, the relatively small sample size may have limited the statistical power of some comparisons. Indeed, the hospitalizations of the FT and FC groups were not significantly different. The rigorous approach to drain management employed at our institution, including frequent fistulography and drain exchange, may not be feasible in all clinical settings. This may result in an apparent worsening of the POPF severity grade as defined by the ISGPF classification system. Indeed, the postoperative course of patients with POPF who did not undergo fistulography was not evaluated. It was possible that a major leak at the pancreaticojejunostomy may not have been visible in the early stages owing to pressure from the contrast and the jejunum. Furthermore, the misclassification of fistulography findings, particularly in cases with encapsulated pseudocysts or poorly positioned drains, could have affected the accuracy of the results. Prospective multicenter studies with larger patient cohorts are needed to validate these findings and explore the long‐term outcomes of jejunal drainage in POPF management. Further research should investigate the cost‐effectiveness of fistulography‐guided drain management and its impact on patient quality of life. Additionally, advancements in imaging techniques and drain technology may enhance the precision and efficiency of POPF management.

## 5 Conclusion

This study showed that the internal drainage of pancreatic juice into the jejunum is crucial for the healing of POPF after PD. Fistulography‐guided drain management effectively optimizes patient outcomes and safety. These findings provide insights into the management of this challenging complication of pancreatic surgery and emphasize individualized, evidence‐based postoperative care.

## Author Contributions

**Yoshihiro Miyazaki:** conceptualization, writing – original draft, methodology, data curation. **Shinji Hashimoto:** data curation, writing – review and editing, supervision, conceptualization. **Osamu Shimomura:** data curation, conceptualization, investigation, supervision, writing – review and editing, formal analysis. **Hiromitsu Nakahashi:** writing – review and editing, data curation. **Manami Doi:** data curation, writing – review and editing. **Kazuhiro Takahashi:** writing – review and editing, data curation. **Jaejeong Kim:** data curation, writing – review and editing, visualization, software. **Shuntaro Tsukamoto:** data curation, writing – review and editing. **Kinji Furuya:** writing – review and editing, data curation. **Yohei Owada:** data curation, writing – review and editing. **Koichi Ogawa:** writing – review and editing, data curation. **Yoshimasa Akashi:** data curation, writing – review and editing. **Tsuyoshi Enomoto:** writing – review and editing, data curation. **Tatsuya Oda:** data curation, writing – review and editing, supervision, conceptualization, funding acquisition, validation, project administration.

## Ethics Statement

This study was approved by the Institutional Review Board of the University of Tsukuba Hospital (R01‐030).

## Consent

Informed consent was obtained from all participants included in the study.

## Conflicts of Interest

The authors declare no conflicts of interest.

## Supporting information

Figure S1

## Data Availability

The data that support the findings of this study are available from the corresponding author upon reasonable request.
