# Supplementary material for: Promoting Pancreatic Fistula Healing After Pancreaticoduodenectomy Through Internal Drainage of Pancreatic Juice Into the Jejunum
Source: World J Surg. 2025 Jul 12;49(8):2228–35. doi: 10.1002/wjs.12679 (PMC12338440; doi:10.1002/wjs.12679)

**Supplementary Figure a.** Overview of the study population. **b**. The workflow of postoperative drain management.


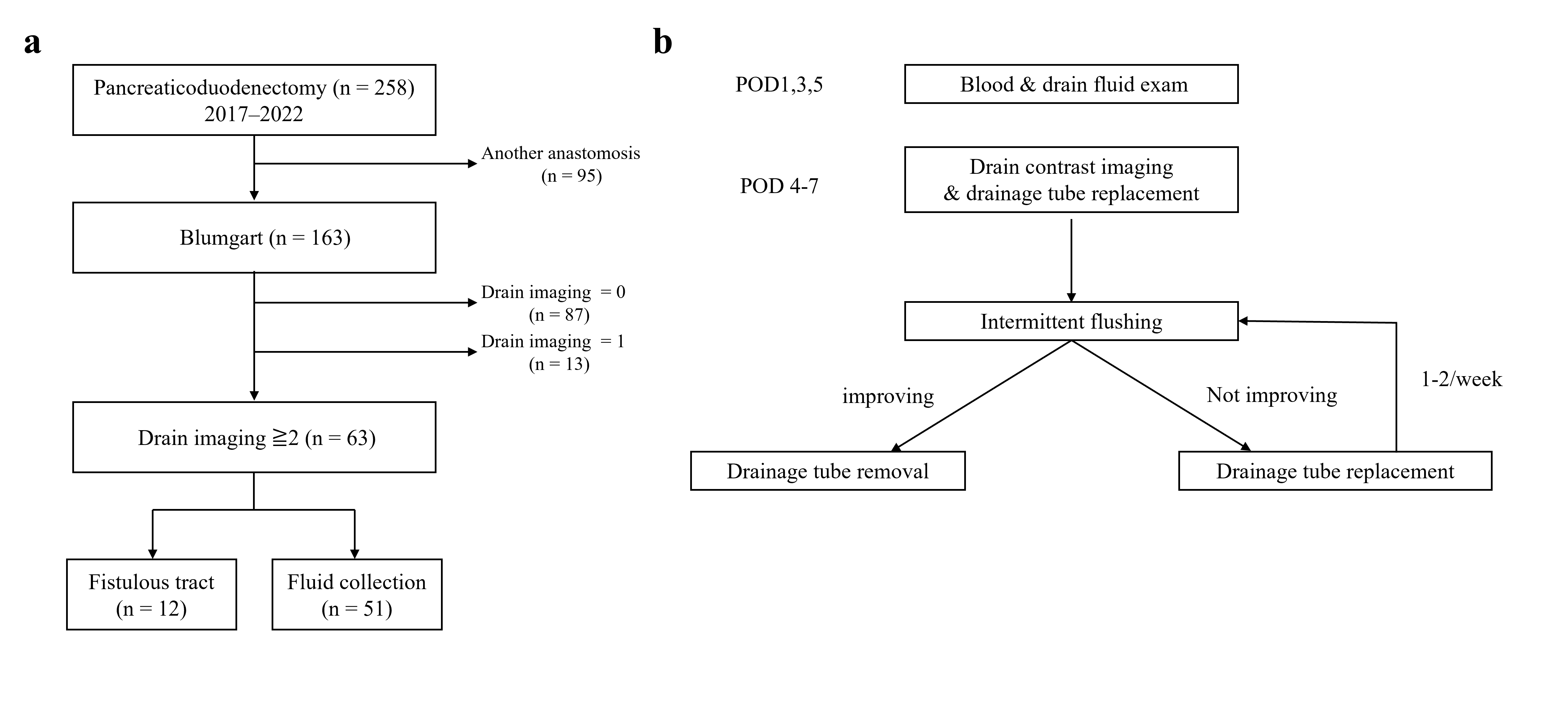

Supplement: Supplementary file 1 — Figure S1 [file WJS-49-2228-s001.docx]
